# Programmable Paper-Based Microfluidic Devices for Biomarker Detections

**DOI:** 10.3390/mi10080516

**Published:** 2019-08-02

**Authors:** Veasna Soum, Sooyong Park, Albertus Ivan Brilian, Oh-Sun Kwon, Kwanwoo Shin

**Affiliations:** Department of Chemistry, Institute of Biological Interfaces, Sogang University, Seoul 04107, Korea

**Keywords:** paper-based microfluidic device, flow control, droplet actuation, multi-step assay, biomarker detection, digital microfluidic device

## Abstract

Recent advanced paper-based microfluidic devices provide an alternative technology for the detection of biomarkers by using affordable and portable devices for point-of-care testing (POCT). Programmable paper-based microfluidic devices enable a wide range of biomarker detection with high sensitivity and automation for single- and multi-step assays because they provide better control for manipulating fluid samples. In this review, we examine the advances in programmable microfluidics, i.e., paper-based continuous-flow microfluidic (p-CMF) devices and paper-based digital microfluidic (p-DMF) devices, for biomarker detection. First, we discuss the methods used to fabricate these two types of paper-based microfluidic devices and the strategies for programming fluid delivery and for droplet manipulation. Next, we discuss the use of these programmable paper-based devices for the single- and multi-step detection of biomarkers. Finally, we present the current limitations of paper-based microfluidics for biomarker detection and the outlook for their development.

## 1. Introduction

Because of cost, disposable, paper-based microfluidic devices have become an attractive tool for point-of-care testing (POCT) and medical screening in the developing world. Paper-based, continuous-flow microfluidic (p-CMF) devices were first invented in 1949 by Müller and Clegg [1]; however, no proof of concept for the use of such devices for portable, onsite detection in biosensing applications was reported until 2007 when Whitesides’ group published their report [2]. Not only are p-CMF devices cost-effective and eco-friendly, they can also transport fluid samples via capillary action. Because capillary action in a paper channel is passive, it limits the flexibility of fluid handling. Being the focus of research by many academics, p-CMF devices were soon developed so that they could be programmed to transport fluid samples to target locations within a set time. These programmable p-CMF devices can automatically transport fluid samples in sequence for the whole of detection protocols. Therefore, they can be used in a wide range of analytical assay applications involving both single- and multi-step detection protocols.

Another type of paper-based microfluidic device is the paper-based digital microfluidic (p-DMF) device, which was introduced by Shin’s group [3] and Wheeler’s group [4] in 2014, even though the first DMF had been reported by Pollack et al. in 2000 [5]. P-DMF devices transport a fluid sample (droplet) by using the electrowetting on dielectric (EWOD) technique in which a droplet is actuated by applying an electric field to electrode arrays coated with a hydrophobic dielectric layer [6,7]. Although p-DMF devices are based on the same principle as other DMF devices, they have advantageous features such as portability and the capability to deliver, delay, merge, and split small-volume samples. Because p-DMF devices are inexpensive and easy to fabricate, they are more suitable for POCT than DMF devices fabricated on plastic films [8,9,10], printed circuit boards (PCBs) [11,12], and glass substrates [13,14] are. Since the initial reports on p-CMF and p-DMF devices, they have become attractive research topics and have been developed for real-world applications in biomarker detection.

Easy, low-cost fabrication is a major advantage of paper-based microfluidic devices [15]. The p-CMF is commonly fabricated by patterning a hydrophobic material (fluid barrier) on the surface of hydrophilic paper [1,2,16,17,18] and cutting out a channel from the hydrophilic paper [19,20,21,22]. The paper channel of the p-CMF device is inherently a porous matrix that provides continuous capillary action for fluid transport. The capillary action on the paper surface can be controlled by way of physical and chemical modifications [23,24,25]. Through control of the capillary action, the p-CMF device can be programmed for challenging detection protocols to manage the fluid flow better [25,26]. On the other hand, p-DMF devices are fabricated by patterning conductive electrode arrays on paper substrates and then coating the electrode arrays with a hydrophobic-dielectric layer [3,4]. Because the operation of a p-DMF device is based on the EWOD technique, an electrical switching system and a control software interface are needed to program the fluid transport [27,28].

As analytical platforms, paper-based microfluidic devices are commonly used to transport fluid samples and to store chemical reagents for the colorimetric and electrochemical detection of target analytes [29,30,31,32]. The traditional p-CMF is limited by low sensitivity and by difficulty in achieving multi-step assays with automatic processes [25]. Furthermore, p-DMF devices still require a portable electrical switching system and a controlled software interface for the assay protocols [33]. Programming the fluid transport (delay, mix, merge, split, valve, and sequential fluid delivery) in the p-DMF device provides flexibility of fluidic manipulation and opens a wide range of analytical assays for a better detection result. 

**Figure 1:**
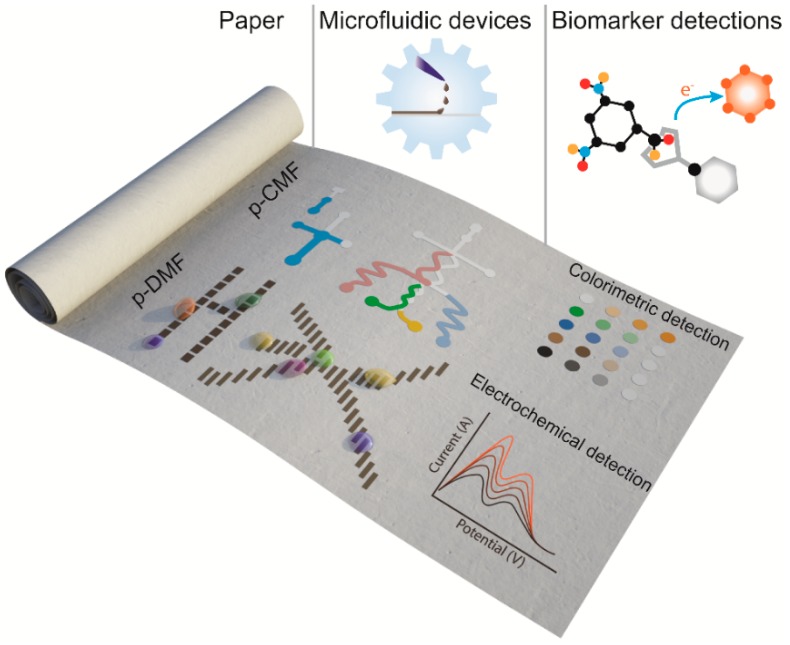
Schematic illustration of a programmable microfluidic device (paper-based continuous-flow microfluidic (p-CMF) and paper-based digital microfluidic (p-DMF)) fabricated on a sheet of paper. Various developed methods can be used to program these devices to manage a fluid sample for high-sensitivity and multi-step assay detection of biomarkers at point-of-care (POC).

In this review, we describe the recent advances in programmable paper-based microfluidic devices from their fabrication methods to their applications for biomarker detection, as schematically shown in Figure 1. For p-CMF and p-DMF devices, we present the methods used for their fabrication and the techniques used to control fluid transport in a programmable way. We give some examples of lab-on-a-chip applications using programmable paper-based microfluidic devices. We end this review with a discussion of the current limitations on and the future aspects for the use of programmable paper-based microfluidic devices in real-world applications.

## 2. Fabrication of p-CMF Devices

The fibrous cellulose network in a filter, chromatography paper, and nitrocellulose paper allow fluid to flow by capillary action in a paper matrix. The fluid flow in the paper is mainly influenced by the cohesive and the adhesive forces that are dominated by the surface tension of the fluid and the surface chemistry of the cellulose fiber network, respectively. A paper-based guided channel for fluid flow can be fabricated by turning a sheet of paper into a paper-based microfluidic platform that can be used to manipulate the fluid sample. The common methods used for the fabrication of paper-based microfluidic devices, such as patterning a hydrophobic barrier, cutting out a channel, layer-by-layer assembly, and laser treatment, can be found in various review papers [15,23,34]. These methods allow paper-based microfluidic devices to be fabricated at low-cost and in a simple way. 

**Figure 2:**
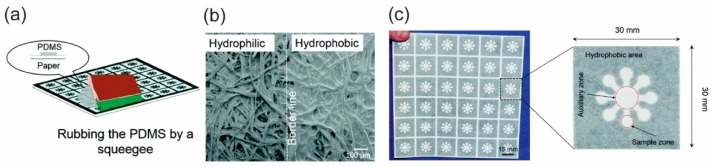
Patterning hydrophobic barriers on paper for fabricating p-CMF devices. (**a**) Screen-printing of polydimethylsiloxane (PDMS ink for patterning PDMS-hydrophobic patterns, (**b**) scanning electron microscope (SEM) images of paper (left) and PDMS-paper (right), and (**c**) fabricated p-CMF devices. Reprinted with permission from the authors of [36]. Copyright 2015 The Royal Society of Chemistry.

Patterning a hydrophobic fluid barrier in a fibrous cellulose network, which was introduced in 1949 by Müller and Clegg [1], was the first method used to fabricate paper-based microfluidics. Creating a hydrophobic fluid barrier involves two principles: physically blocking the porous networks in the paper, and chemically modifying the surfaces of the cellulose fibers in the paper. To block the pores in the paper physically, one has to deposit a hydrophobic material into the matrix of the paper to create fluidic barriers (Figure 2) [15,23]. Paraffin film is a pre-made, low-cost material that is widely used in laboratories to shield containers so as to avoid leakage. Because paraffin is hydrophobic, it can be used to create the hydrophobic barriers needed for the fabrication of p-CMF devices. Depending on the design of the microfluidic channel, one cuts paraffin out from its film and hot-laminates it so that it melts on the surface of the filter paper. The melted paraffin penetrates into the porous network in the paper to create hydrophobic barriers, and the area unoccupied by paraffin remains as a hydrophilic channel; as a result, a p-CMF device can be fabricated [16,35]. 

Hydrophobic wax is a common material used in the fabrication of p-CMF devices because it is cheap and printable. It can be printed by using a wax printer to generate wax patterns on the surface of paper [17,37,38,39]. After patterning, the printed wax has to be heated at about 100 °C to melt so that it can penetrate the paper matrix to block the porous network in the paper. For the fabrication of p-CMF devices, polydimethylsiloxane (PDMS) and polymethylmethacrylate (PMMA) can be used to block the porous networks in paper [36,40]. An aqueous solution of PDMS and PMMA can flow into the matrix of paper immediately after screen-printing; however, the printed patterns require a post-treatment to remove solvents to obtain hydrophobicity. PDMS patterns have to be cured by drying at about 120 °C while PMMA patterns can be left to dry in the ambient. Titanium oxides (TiO_2_) have been used as hydrophobic agents to block the pores in paper [41]. They were formulated as aqueous ink and deposited into the paper by direct handwriting with a correction pen. Another method, chemical surface modification, is performed by introducing a new material to bind to the cellulose paper chemically, thus forming hydrophobic surfaces. The common materials used in the modifications are alkyl ketene dimer (AKD) [42,43] and commercial permanent marker ink [18,44,45]. In the fabrication of p-CMF devices, Inkjet printing and pen-plotting are used for patterning AKD and the permanent marker ink, respectively, onto the surface of the paper [42,43,44]. Trichlorosilane (TCS) is deposited on paper by using chemical vapor deposition (CVD) to create fluidic barriers in p-CMF devices [46]. 

**Figure 3:**
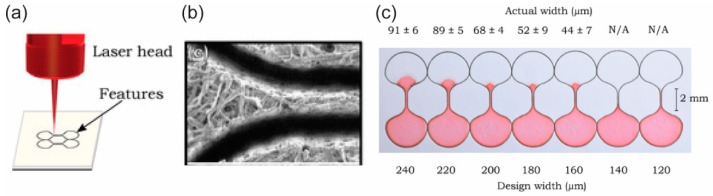
Cutting and shaping paper in the fabrication of p-CMF devices. (**a**) The use of a laser beam to cut and shape paper to form a large paper channel, (**b**) SEM image of the paper channel formed by using the laser, and (**c**) prepared p-CMF devices with various channel widths. Reproduced with permission from the authors of [20]. Copyright 2018 under the terms and conditions of the Creative Commons Attribution.

Cutting a piece from porous paper, such as a filter or chromatography paper, is a straightforward method for fabricating paper-based microfluidic devices. It does not require any external material to be deposited in the paper matrix as the above methods do because the porous networks in the paper end at the cutting edge (Figure 3b). The part of the paper cut from the paper sheet is directly used as the microfluidic material (Figure 3c). This method mainly relies on cutting with a blade [21,22] or a laser, for example [20,47]. Cutting the paper with a blade is called the contact-cutting mode and can be performed manually by using scissors or a blade with a mask. The cutting can also be done digitally by using a digital cutting plotter with its blade. The cutting resolution depends on the sharpness of the blade and the toughness of the paper. On the other hand, cutting the paper with a laser is called the non-contact cutting mode and provides fast, high quality cutting with programmable control (Figure 3a). 

**Figure 4:**
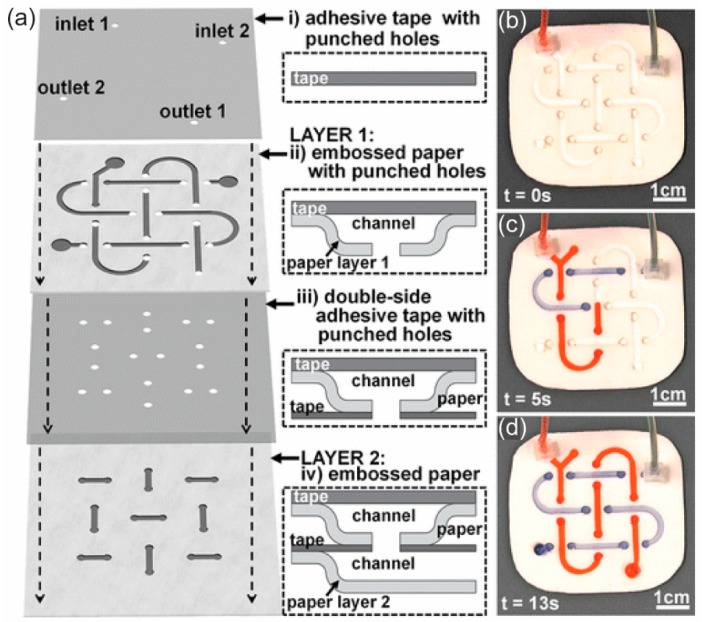
(**a**) Fabrication of a 3D p-CMF device by assembling components such as adhesive tape and embossed paper. (**b**) The fabricated 3D p-CMF device before fluidic loading and (**c**) after 5 s of fluidic loading. (**d**) The flow of the fluid at the ends of the channels. Reprinted with permission from the authors of [48]. Copyright 2014 American Chemical Society.

Paper-based microfluidic devices can be fabricated by assembling the cut paper, the paper or the adhesive film, the paper to form a three-dimensional (3D) channel (Figure 4). Stacking, origami, and lamination can be used to assemble each layer of the microfluidic device [48,49,50,51,52,53]. This fabrication method relies on the abilities to pattern a hydrophobic fluid barrier and to use a cutting method to generate each layer before the layers are assembled together. The assembling of the layers of the fabricated microfluidic device in the stacking and the origami methods requires a holder to ensure that the layers stay in contact with one another. The microfluidic devices fabricated by using the lamination technique do not require a holder because each layer can bind together due to the adhesive material coated on the laminating films. Molding is another way to fabricate a paper channel. In this method, a mold is created and filled with a mixture of cellulose powder and water [54]. A paper channel with cellulose networks based on the shape of the mold is generated after drying. 

**Figure 5:**
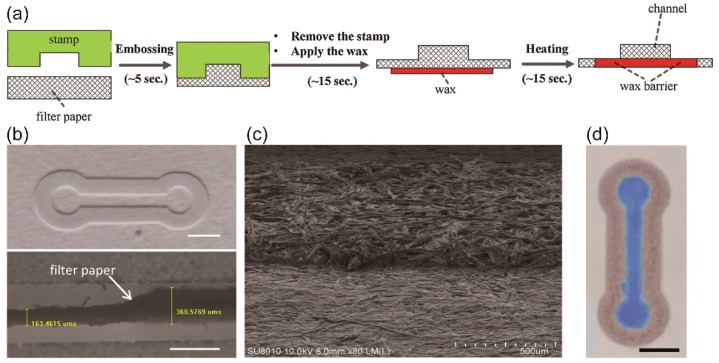
(**a**) Schematic of the fabrication process, including embossing, for the p-CMF device. (**b**) Images of the top (top) and cross-sectional (bottom) views of the embossed paper channel. (**c**) SEM image of the boundary of the embossed paper channel before applying wax. (**d**) p-CMF device after applying the wax and loading it with blue ink. Scale bars represent 5 mm. Reprinted with permission from the authors of [55]. Copyright 2018 Elsevier B.V.

Embossing is a relatively new and low-cost fabrication method for p-CMF. Because paper is soft and porous, it can be compressed to form any desired geometry. Using a compressor to apply sufficient pressure to the paper with a stamp, researchers can rapidly form paper channels (Figure 5a–c). The paper channel can either be a compressed or molded area [48,55,56]. However, the pristine channel requires hydrophobic barriers to work as guided barriers (Figure 5d). The hydrophobic barriers can be created by silanization on the compressed channels [48] and applying wax to the boundaries of the molded channels [35,55,56]. During the embossing process of a hot-embossing method, hydrophobic barriers and paper channels can be made, so no post-treatment is required for the fabrication [56].

**Figure 6:**
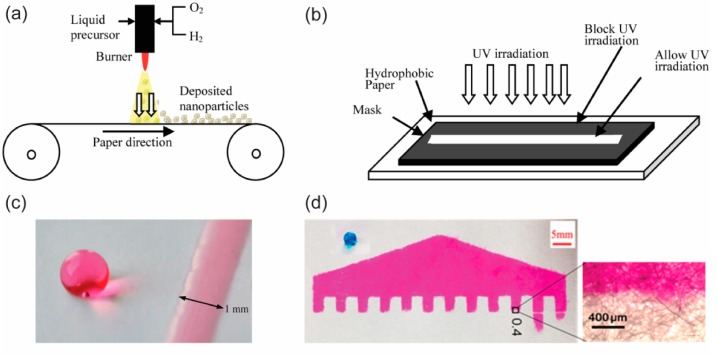
(**a**,**b**) Schematic showing the use of a laser to irradiate a hydrophobic surface to fabricate a p-CMF device. (**c**) Water droplet on a hydrophobic surface and the spreading of water on a hydrophilic surface that had been subjected to UV irradiation. Reprinted with permission from the authors of [58]. Copyright 2014 American Chemical Society. (**d**) A typical design of a fabricated p-CMF device, and a high magnification image of the boundary between the hydrophobic and the hydrophilic surfaces. Reprinted with permission from the authors of [62]. Copyright 2018 American Chemical Society.

A laser beam can cause a hydrophobic surface to become a hydrophilic surface. Based on the hydrophilic-hydrophobic contrast principle in the fabrication of p-CFM devices, one can use laser light to irradiate hydrophobic paper to produce hydrophilic channels in the surface of that paper (Figure 6). First, pristine paper has to be coated with a hydrophobic agent, for instance, octadecyltrichlorosilane (OTS) n-hexane [57], TiO_2_ [58,59], a photopolymer [60,61], or a hydrophobic so-gel [62,63], to generate hydrophobic paper. Once the hydrophobic paper has been made, a designed area on the hydrophobic surface can be exposed to laser irradiation to create hydrophilic channels. The hydrophilic channels allow fluid to flow while the remaining hydrophobic area functions as a fluid barrier. Summary of some published fabrication methods of p-CMF devices is shown in Table 1. 

## 3. Fabrication of p-DMF Devices

Digital microfluidic devices actuate a fluid droplet based on the EWOD technique, which requires a set of conductive electrode arrays, a dielectric layer, and a hydrophobic layer. The conductive electrode array allows the applied voltage to induce an electrostatic force at the interfaces between the dielectric layer and conductive liquid while the hydrophobic layer minimizes the surface energy and the surface friction. The characteristics of the conductive electrode arrays and the hydrophobic-dielectric layer are important parameters that influence the performance and the robustness of a DMF device. We should note that the fabrication cost of those components must be low if the devices are to be used for POCT. Because DMF devices can be fabricated on paper substrates, the so-called p-DMF devices, they should be cheap, disposable, and suitable for low-resource laboratories.

**Figure 7:**
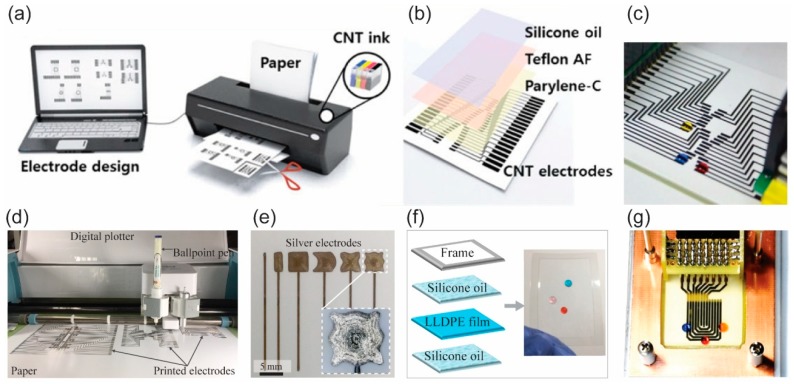
Fabrication of p-DMF devices. (**a**) Patterning of conductive electrode arrays for a p-DMF chip by Inkjet printing of CNT ink on paper. (**b**) Schematic illustration of the components of a p-DMF device: CNT electrodes, Parylene-C dielectric layer, Teflon AF hydrophobic layer, and slippery silicone oil layer. (**c**) A fabricated p-DMF chip. Adapted with permission from the authors of [3]. Copyright 2014 WILEY-VCH Verlag GmbH & Co. KGaA, Weinheim. (**d**) Printing of conductive electrode arrays for a p-DMF chip on paper by using a ballpoint pen with AgNP ink and a digital plotter. (**e**) Printed electrodes, (**f**) a prepared dielectric layer modified with silicone oil, and (**g**) a p-DMF chip. Reproduced with permission from the authors of [69]. Copyright 2019 under the terms and conditions of the Creative Commons Attribution.

The fabrication of a p-DMF device starts from the patterning of conductive electrode arrays on a paper substrate. The patterning of conductive electrode arrays can be done by using photolithography or an etching method, which requires clean-room facilities. For simple, low-cost patterning of conductive electrode arrays for p-DMF devices, Shin’s group introduced the Inkjet printing method [3,28]. They printed carbon nanotube (CNT) ink by using an office Inkjet printer to pattern conductive CNT electrodes on photo paper (Figure 7a). Fobel et al. also used the Inkjet printing method for patterning conductive electrode arrays on photo paper [4]. Their conductive electrode arrays were printed using silver nanoparticle (AgNP) ink, so they could obtain electrodes with higher conductivity compared to the ones printed from CNT ink. The Inkjet-printed electrodes are thin (about submicron to a few microns thick), which is suitable for thin dielectric layer coating, the next step in the fabrication of p-DMF devices. Another printing method, screen printing, can be used for patterning conductive electrodes on paper [65,66]. By using this method with silver-based and carbon-based ink, Yafia et al. printed multiple conductive electrode arrays on paper [65]. Screen-printed electrodes are generally thick (≥10 μm) because the ink used for printing has high viscosity. With high-pressure air, Abadian and Jafarabadi-Ashtiani generated conductive electrode arrays on paper by spraying graphite onto paper that had been covered with a mask [67]. Jafry et al. introduced a micro-syringe pump to dispense AgNP ink for patterning conductive electrode arrays on filter paper [68]. A contact printing method using a ballpoint pen with a digital plotter was recently proposed and used for patterning AgNP electrode arrays on photo paper (Figure 7d,e) [69]. That method provides an affordable way for printing in a programmable manner. When electrodes are printed in this way, their thicknesses are roughly submicron, and they have high electrical conductivity.

After the conductive electrode arrays have been fabricated, a dielectric layer and a hydrophobic or slippery surface are required to make a complete p-DMF device (Figure 7b–g). The material used for the dielectric layer has to have a high breakdown voltage to avoid dielectric malfunction when a high voltage is applied during operation. Parylene-C, a reliable dielectric material for use in electronics, is commonly used as the dielectric layer for p-DMF devices [3,4,28]. It can be deposited on the printed electrode arrays by using chemical vapor deposition (CVD). Another material, a PDMS thin film, can be prepared by spin coating and used as the dielectric layer for a p-DMF chip [68]. For an easy, low-cost method without the need for clean-room facilities, researchers have proposed some affordable materials, such as parafilm [65], adhesive tape [66], and commercial food wrap [69], for use as dielectric layers. Because most of these dialectic materials are hydrophilic or have low hydrophobicity, for better EWOD performance, a hydrophobic and/or slippery layer is required. Teflon is a common hydrophobic material used for hydrophobic coating while silicon oil is used for generating slippery surfaces [4,69,70]. Summary of some published fabrication methods of p-DMF devices is shown in Table 2.

## 4. Strategies for Programming the Delivery of a Fluid Sample in p-CMF Devices

Delivery of fluid samples in the p-CMF device is a passive process and results from capillary flow or wicking flow inside the fibrous cellulose network. This passive flow in the p-CMF device can be described by using the Lucas Washburn equation, which was derived to describe capillary flow in a one-dimensional model in cylindrical tubes. In that equation, the flow distance (*x*) is proportional to the square root of time (*t*^1/2^):

(1) $$x=\sqrt{\frac{\gamma r\cos\left(\theta \right)t}{2ŋ}}$$

where *γ*, *θ*, and *ŋ* are the fluid surface tension, the contact angle of the fluid with the channel wall, and the fluid viscosity, respectively; *r* and *t* are the pore radius of the paper and time, respectively. Based on the Lucas Washburn equation, the physical structure and the surface chemistry are the critical microfluidic parameters that control the speed of fluid flow in the p-CMF device. With these concepts, researchers have developed a variety of programmable p-CMF devices by using various methods to control these parameters. Some of the excellent methods, such as variation of the channel dimensions (width, and depth), control of the permeability of the paper, use of pattern fluid-flow regulators, and control of the surface chemistry of the paper channels, have been used to manipulate fluid sample delivery in p-CMF devices. 

**Figure 8:**
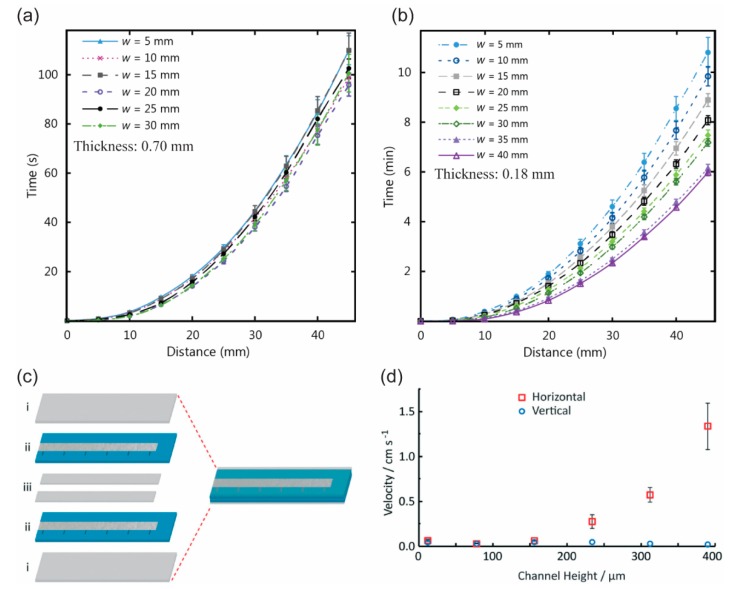
Influence of the geometries of the p-CFM channels on fluidic flow behavior. (**a**,**b**) Thickness and width of paper channels, (**c**,**d**) height of channel (the gap between the top and the bottom surface). (**a**,**b**) Reprinted with permission from the authors of [72]. Copyright 2016 under the terms and conditions of the Creative Commons Attribution. (**c**,**d**) Reproduced with permission from the authors of [75]. Copyright 2018 The Royal Society of Chemistry.

When the channel width is varied, several parameters must be explored to explain the wicking flow of the fluid in open paper channels. In a paper channel that is thick (~0.70 mm) and has a straight boundary, wicking flow shows no changes when the width of the channel is varied (Figure 8a) [71,72]. However, wicking flow decreases in a thinner channel (0.18 mm) when the width of the channel becomes smaller (Figure 8b) [72]. The decrease in flow speed in a thin channel can be explained by the edge effect of the cut channel where the edges hinder the flow. In a flow effect similar to that for a thin channel with cut boundary, a hydrophobic boundary channel enables a faster flow speed in a thicker channel with a larger width (horizontal orientation) (Figure 8c,d) [71,73,74,75]. When the channel width and height are increased, the edge effect is significantly reduced over the total area of the channel, which allows faster flow. Another method to manipulate the flow in the hydrophobic boundary channel is to carve the channel either longitudinally or perpendicularly, thus accelerating or de-accelerating the flow, respectively [76]. The longitudinally crafted channels create a flow path for fast laminar flow while the perpendicularly crafted channels resist the flow.

**Figure 9:**
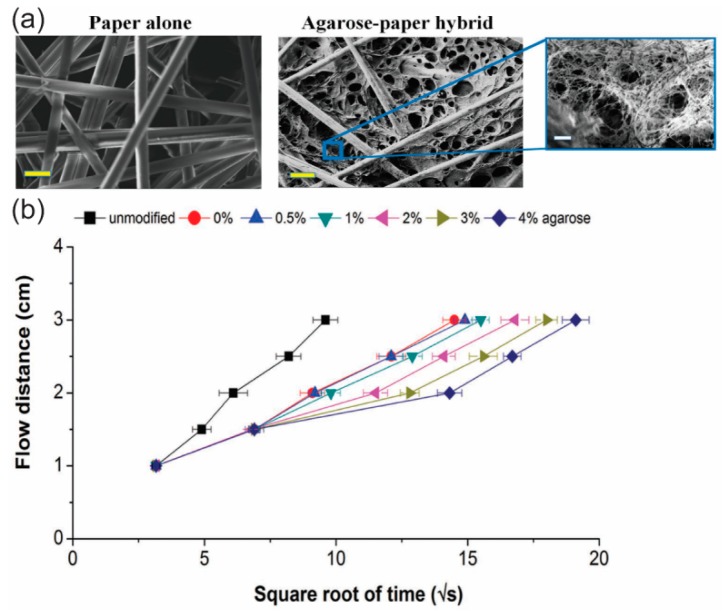
Regulation of the fluid flow in a p-CMF device by controlling the permeability of the paper channel. (**a**) SEM images of paper (left) and of agarose-paper (right). (**b**) Fluid flow distance versus square root of time in paper channels with and without treating with various concentrations of agarose. Reprinted with permission from the authors of [79]. Copyright 2016 WILEY-VCH Verlag GmbH & Co. KGaA, Weinheim.

Depending on the capillary force, the characteristics of liquid flow in a paper-based channel are influenced by the permeability of the porous paper. Hydrophobic materials can be used to reduce the pore size of the paper so that the permeability is minimized. Wax can be melted so that it can penetrate the matrix of the porous paper to block some pores, or at least minimize their size, because the permeability of the paper depends on the amount of wax deposited in the paper. As the permeability of the paper channel is reduced, the flow speed of the liquid in the channel is decreased [77,78]. Another material, agarose, can be used to control the permeability of porous paper (Figure 9a). Agarose can penetrate the matrix of the porous paper so that the pore size becomes smaller (600 nm to 250 nm) with increasing concentration of agarose (0.5% to 4.0%) [79]. The smaller pore size leads to reduced permeability, so the flow in the paper matrix is slower (Figure 9b).

**Figure 10:**
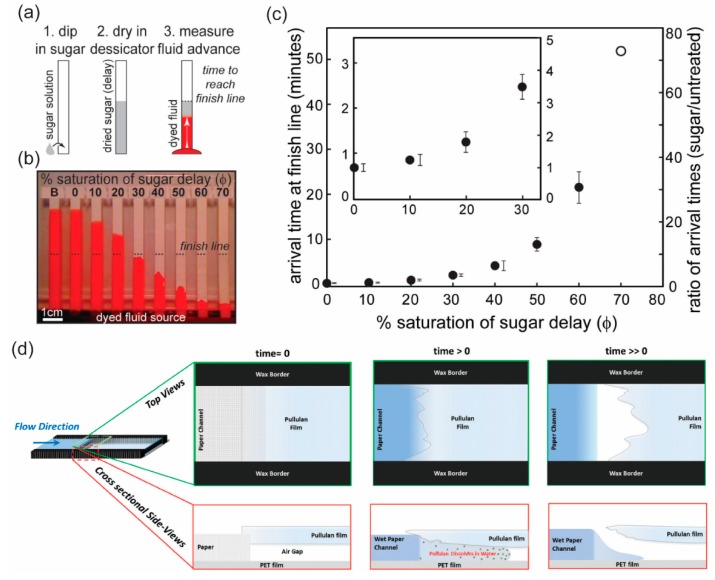
Delay of fluidic flow by incorporating a dissolvable material in a p-CFM device. (**a**) Schematic of a method for preparing a p-CMF device for which time delay is controllable. (**b**) Images of fluidic flow distances at specific times in paper channels modified with various concentrations of sugar. (**c**) Arrival time of fluid at the ends of channels versus the concentration of sugar used in the paper channels. Reproduced with permission from the authors of [81]. Copyright 2013 The Royal Society of Chemistry. (**d**) The pullulan film works as a dissolvable bridge to shut off the flow of liquid when a certain amount of liquid has passed through the bridge. Reproduced with permission from the authors of [83]. Copyright 2014 The Royal Society of Chemistry.

Dissolvable materials have been used to delay the flow of fluid in p-CMF devices. Dissolvable sugars (trehalose, sucrose) have been used to block temporarily the pores in the paper channel and enhance the viscosity of the fluid [80,81]. During flow, the fluid dissolves the sugar in the paper channel. The dissolution of sugar delays the flow and enhances the viscosity of the fluid. With increasing amount of sugar in the paper channel, the fluid flow can be dramatically delayed (Figure 10a–c). Because of this effect, dissolvable materials have been used to control the volume of the aqueous sample to be delivered by the p-CMF device. Houghtaling et al. formulated dissolvable bridges made of sugar (trehalose) and used them to automate the delivery of fluid samples [82]. Their dissolvable bridge was mounted between the source pad and the main channel. The volume of fluid that passes the dissolvable bridge is defined by the cross-sectional area and the composition of the bridge and by the choice of the feeder material. Jahanshahi-Anbuhi et al. used water-soluble pullulan films as valves in p-CMF devices to shut off the flow after the pullulan film had been dissolved because a sufficient volume of liquid had passed through it (Figure 10d) [83].

**Figure 11:**
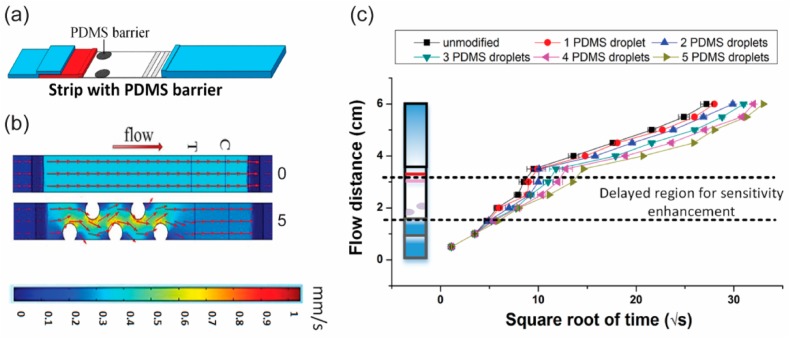
Regulation of the fluid flow in a p-CMF device by using hydrophobic patterns. (**a**) Schematic for the modification of a p-CMF device with PDMS barriers, (**b**) simulation of the flow of fluid in an unmodified and a modified channel, and (**c**) flow distance versus square root of time in the p-CMF devices unmodified and modified with different numbers of PDMS droplets (barrier). Adapted with permission from the authors of [86]. Copyright 2016 American Chemical Society.

Hydrophobic materials are commonly patterned in the paper channels to regulate the fluid flow in p-CMF devices. The hydrophobic patterns in the paper channel act as obstacles to delay the flow of fluid (Figure 11). Wax pillars were printed on nitrocellulose membrane by Rivas et al. to delay the flow of a fluid sample in the channel [84]. The wax pillars were printed onto the nitrocellulose membrane by using a wax printer; this was followed by heating at 110 °C for 90 min. Using this method, they could slow the flow to 0.18 × 10^−3^ m/s. Preechakasedkit and co-workers also printed wax as line patterns onto a nitrocellulose membrane channel by using a wax printer [85]. With their design, they could delay the sample delivery time in their channel by approximately 11 s. PDMS patterns were also used as fluidic barriers to slow fluid flow (Figure 11a,b) [86]. The PDMS patterns were created by using a pipette to drop manually 0.1 μL of PDMS solution onto a nitrocellulose membrane. With five patterns, the delivery of the fluid sample could be delayed by 97.2 ± 0.4 s (Figure 11c). He et al. used a laser direct-write (LDW) method to induce a photopolymer to create solid barriers in a paper channel [87]. When the speed of laser direct writing was slower, the polymer patterns created for use as fluidic barriers were thinner. In this way, the fluid flow can be significantly decreased. 

**Figure 12:**
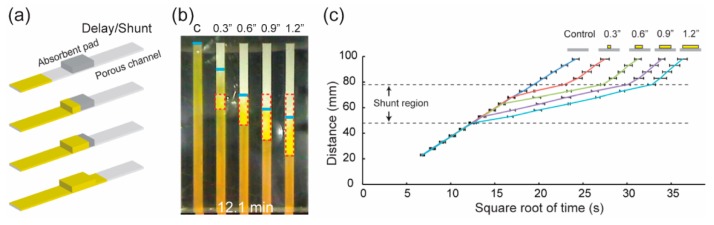
Delay of the fluid flow in a p-CFM device by using absorbent pads. (**a**) Schematics of the flow of fluid in a paper channel modified with an absorbent pad, and (**b**) images of fluid’s travel in paper channels modified with absorbent pads of various lengths. (**c**) Flow distance versus square root of the time the fluid is flowing through paper channels modified with absorbent pads of various lengths. Reprinted with permission from the authors of [88]. Copyright 2013 American Chemical Society.

Absorbent materials can drag fluid into their matrices through capillary action. The absorbent pads made of porous paper or porous polymer can be used in p-CMF devices to delay the flow of fluid. Toley et al. used paper-based absorbent pads (shunt) mounted on a paper channel to divert liquid flowing in the channel in order to delay delivery of the sample (Figure 12) [88]. By varying the shunt length and thickness, they could achieve 3 to 20 min of time delay (Figure 12b,c). Similar to the above concept, Tang et al. used sponge shunts made of porous polyurethane to decrease the flow rate in a p-CMF device by placing the shunt at a junction between a conjugation pad and a main channel [89]. Akyazi et al. proposed photopolymerized ionogel for delaying the flow of fluid in a p-CMF device [90]. The ionogel solution was deposited on the surface of the paper channel; this was followed by irradiation with UV light for photopolymerization. When the photopolymerized ionogel came into contact with the fluid, it absorbed the fluid into its matrix and swelled. The absorption and the swelling processes delay the time of fluid travel through the paper channel. 

**Figure 13:**
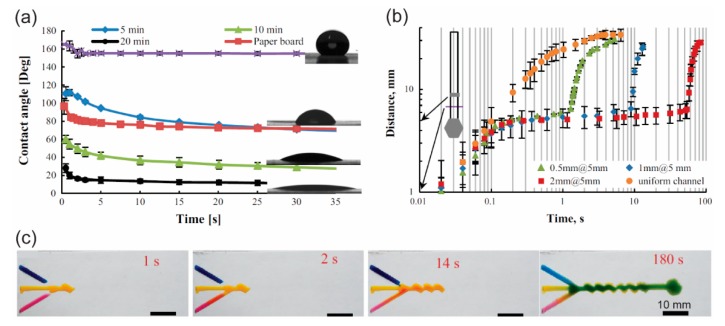
Changing the surface wettability of a paper channel by treating it with UV irradiation to control the flow of fluid in the channel. (**a**) Surface contact angle of a water droplet as a function of time on a paper surface treated with UV irradiation for 0, 5, 10, and 20 min. (**b**) Flow distance of the fluid versus time traveled in paper channels with various geometries due to changes in the wettability of the channel’s surface. (**c**) Sequential fluid delivery in the p-MF device. Adapted with permission from the authors of [91]. Copyright 2016 Springer-Verlag Berlin Heidelberg.

Controlling the surface chemistry of the paper channel is an effective method for programming the delivery of a fluid sample by a p-CMF device. When the hydrophobic surface of paper is exposed to laser light, it can become a hydrophilic surface [59]. If the surface wettability of a microfluidic channel can be controlled, both the capillary action in the channel that allows the fluid to flow and the flow speed of the fluid can be controlled (Figure 13). Songok et al. used UV light to irradiate hydrophobic paper (CA ≈ 160°) to create a hydrophilic surface (CA ≈ 15°) for the fabrication of p-CMF devices (Figure 13a) [58,91,92]. They could vary the time delays of fluid flow by varying the geometry of their paper channels (Figure 13b) [91], and they could create fast capillary flow with speeds up to approximately 9 mm/s by adding a hydrophobic top cover on their paper channels (Figure 13c) [92]. 

**Figure 14:**
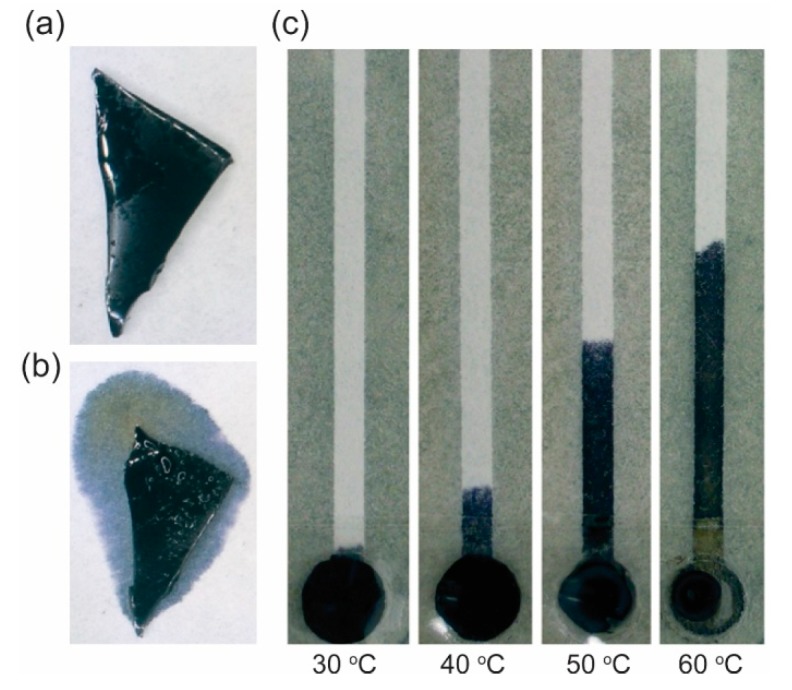
Controlled release of a fluid in a p-CMF device by using a thermoresponsive hydrogel. Fluid (**a**) stored in and (**b**) released from a hydrogel at high temperature. (**c**) Controlling the length of fluid flow in a p-CMF device by heating it at different temperatures. Reproduced with permission from the authors of [93]. Copyright 2015 The Royal Society of Chemistry.

Walji and MacDonald studied the influence of temperature on the wicking flow of fluid in a p-CMF device [72]. They found that the fluid flows faster in a paper channel that has been cured at a higher temperature. The 45 °C fluid took about 8 min to flow through a 45-mm channel while the 15 °C fluid took 11 min. Niedl and Beta controlled fluidic transport in p-CMF devices by using heat [93]. Their paper channels were mounted with thermoresponsive hydrogels (NIPAM-AcAm) that stored fluid (Figure 14). At higher temperature, the hydrogels collapse faster and continuously release fluid into the paper channel at a higher rate (Figure 14b,c). They were able to control the release of fluid by varying the temperature in their paper channels.

## 5. Droplet Manipulations in p-DMF Devices

**Figure 15:**
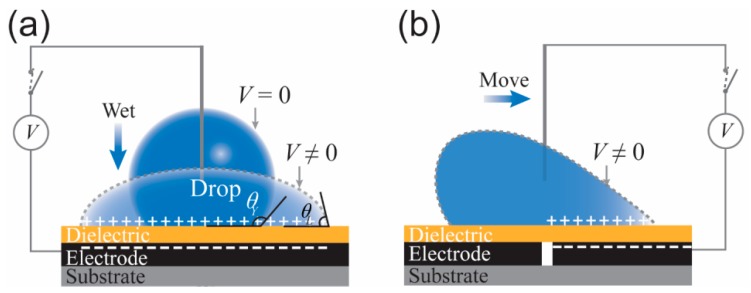
Schematic illustrations of the activation of a fluid droplet on a DMF chip by using the electrowetting on dielectric (EWOD) technique and of droplet wetting when a certain voltage is applied to the system: (**a**) symmetric and (**b**) asymmetric.

Manipulations, such as transporting, mixing, and merging, of fluid droplets on a p-DMF device are controlled by switching off and on the electrical power applied to the conductive electrode arrays. When a certain voltage is applied, a droplet starts to decrease its contact angle (CA) and reaches a new equilibrium, the so-called wetting equilibrium (Figure 15a). This phenomenon can be explained by the Young–Lippmann equation:

(2) $$\cos\theta_{L}(V)=\cos\theta_{Y}(0)+\frac{1}{2\gamma_{lv}}CV^{2},\cos\theta_{L}(V)=\cos\theta_{Y}(0)+\frac{1}{2\gamma_{lv}}CV^{2},$$

where *θ_L_*(*V*) and *θ_Y_*(0) are the CA of a droplet when a voltage *V* and no voltage are applied, respectively. *γ_lv_* is the interfacial tension of conductive liquid (droplet). *C* and *V* are the capacitance of the interface and the applied voltage, respectively. When the symmetry of a wetted droplet is broken, it can be moved to a higher wetting surface by an applied voltage (Figure 15b). 

**Figure 16:**
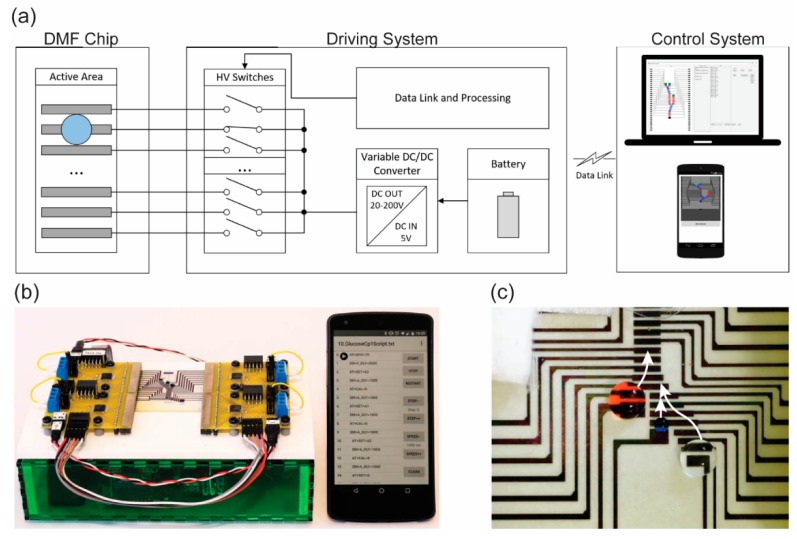
(**a**) Schematic illustration and (**b**) image of the operation system for a p-DMF device. Reproduced with permission from the authors of [27]. Copyright 2017 IEEE. (**c**) A p-DMF chip actuating a droplet by using the operation system. Reproduced with permission from the authors of [28]. Copyright 2017 WILEY-VCH Verlag GmbH & Co. KGaA, Weinheim.

Conventionally, when the electrical power applied to operate a p-DMF device is switched on or off, the switch is operated manually or by using the LabVIEW or the MATLAB software [3,68]. When programmable software is used, the handling of the fluid droplets on a p-DMF device is much easier to control than it is when manual switching is used. However, the above systems lack portability because of the number of required devices, such as power supplies, computers, and Arduino circuit boards. Recently, a wireless, portable control system was built to operate a p-DMF device (Figure 16a) [27,28,69]. The control system is based on an Android smartphone operated by the Android OS, which can be installed by using an application called DMF toolbox (version 1.0) (Figure 16b). When the proposed system was used, a p-DMF chip could manipulate fluid droplets in a simple and programmable way (Figure 16c).

Because electrode arrays of p-DMF devices and connection lines are printed on the same side of the paper, they can interfere with each other during a droplet’s actuation when they overlap the droplet. Without a feedback system, a droplet can exhibit unexpected movement when an automatic control system is used. To solve this problem, researchers proposed better algorithms for eliminating the interferences during the operation of a p-DMF chip [94,95].

## 6. Biomarker Detection by Using Programmable p-CMF Devices

That the p-CMF device can transport a sample without the need for an external device makes it very suitable for use as a POCT device. Programming the fluid transport is the ultimate way to achieve an advanced lab-on-a-chip application for the detection of analytes, and it enables single-step and automated multi-step assay protocols with high detection sensitivity. The high detection sensitivity of the p-CMF device is due to the regulation of physical flow and the time delay of the reaction. Because the samples in the p-CMF devices can be programmed for delivery in sequence to the reaction zone, automated multi-step assay detection is achieved.

**Figure 17:**
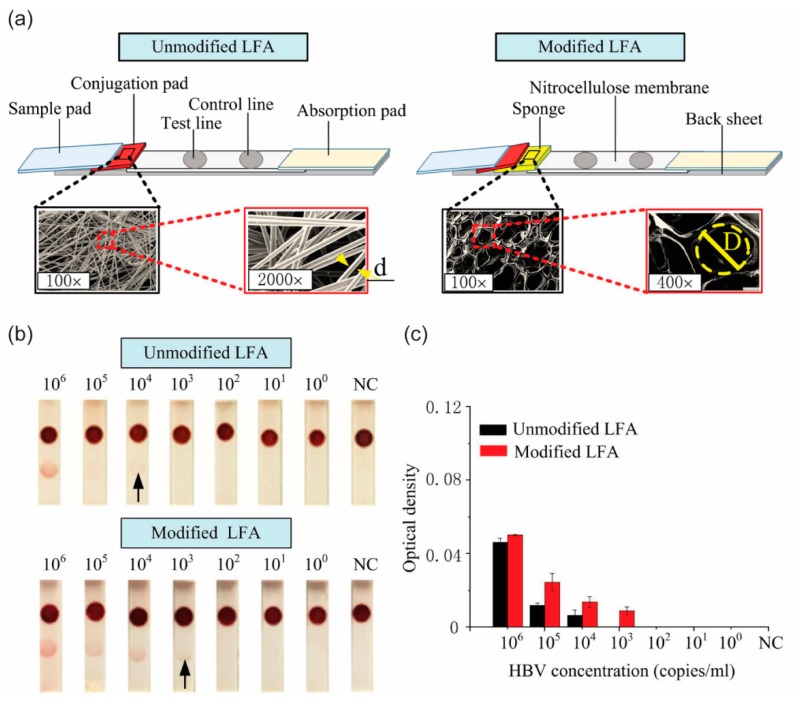
Increasing the detection sensitivity by regulating the flow of the fluid sample in a p-CMF device. (**a**) For lateral flow assay, a sponge was incorporated into a p-CMF device to decrease the flow rate of the fluid sample. (**b**,**c**) Comparison of the colorimetric detection signals of hepatitis B virus (HBV) for the unmodified and the modified p-CMF devices. Adapted with permission from the authors of [89]. Copyright 2017 under a Creative Commons Attribution.

Colorimetric detection using paper-based microfluidic devices is a common method for detecting target analytes [96,97]. Because the result can be read qualitatively and quantitatively by using the naked eye, this technique has emerged as a prime candidate for use in POCT. However, the detection sensitivity in conventional p-CMF devices is relatively low. Some excellent methods [98] to amplify the detection signal in p-CMF devices, such as the use of metal (Ag, Au, Fe_3_O_4_/Au) nanoparticles [99,100,101], catalysts (horseradish peroxidase (HRP), Pt nanocrystals [102,103], and chitosan to modify the paper’s surface [104], have been reported. Moreover, regulating the flow behavior of fluid samples by using a controllable p-CMF device is a straightforward method for enhancing the signal in colorimetric detection (Figure 17). When fluidic barriers, such as wax or PDMS patterns, are introduced into the paper matrix in p-CMF devices, the flow behavior of the fluid sample can change from laminar to turbulent flow, which would improve the sensitivity for detection of analytes by up to threefold [84,85,86]. Absorbent pads (shunts, sponges) have been used for decreasing the flow rate of fluid samples in p-CMF devices to achieve higher signals when detecting nucleic acids [79,86,89]. Designing a p-CMF device to have spatial constrictions of the flow path may lead to a slower flow rate of the fluid sample at the detection zone, thereby allowing more reactants to be bound together; therefore, the signal intensity should be increased [105]. 

**Figure 18:**
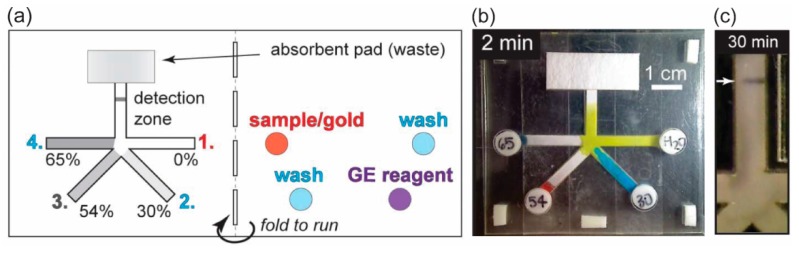
Automated multi-step assay by using a p-CMF device with programmable delivery of the fluid samples. (**a**) Schematic design of a p-CMF for sequential sample delivery in a multi-step assay protocol for detection of malaria. (**b**) Image of sequential sample delivery in the p-CMF device and (**c**) colorimetric detection of malaria, which was achieved by using the programmable p-CMF device. Adapted with permission from the authors of [81]. Copyright 2013 The Royal Society of Chemistry.

Most conventional p-CMF devices have the limitation that they cannot automatically transport fluid samples in sequence while most assay protocols for detecting biomarkers require multi-step reactions. To detect those biomarkers using multi-step assay protocols, researchers have developed methods for programming sequential sample delivery in p-CMF devices without requiring valves and pumps. Without the need for these additional devices to control the sample transport, programmable p-CMF devices have become attractive microfluidic platforms for biomarker detection in POCT. Absorbent pads made of cellulose can be placed on the paper channel to divert fluid into it and delay fluid flow in a p-CMF device [88]. If the thickness and the length of the absorbent pads are increased, the fluid flow delay can be increased from 3 min to 20 min. With this control technique, transport of fluid samples in sequence to a detection zone has been realized for the detection of the malaria protein *Pf* HRP2. By varying the amount of sugar (sucrose) dissolved in the channel of a p-CMF device, the time delays of fluid sample delivery could be varied from minutes to nearly an hour (Figure 18) [81]. Because a higher content of sugar in the channel provided a lower fluid-flow speed (longer delay time), the sugar contained in the paper-based channels was varied to allow fluid samples to be delivered to a detection zone in sequence (Figure 18a,b). A multi-step assay for detection of malaria was successfully performed by using a p-CMF device (Figure 18c). A controllable p-CMF device was fabricated by shining laser light onto a filter paper coated with a photo-polymerizable polymer to create a solid barrier. In this p-CMF device, the fluid flow could be delayed from a few minutes to over half an hour by varying the number and the thicknesses of the polymer barriers [87]. This fabricated p-CMF device could be programmed to deliver fluid samples and reagents sequentially for automated multi-step detection of C-reactive protein (CRP). A p-CMF device was fabricated by layering dry pullulan films containing reagents on paper and was successfully used for sequential sample deliveries for multi-step assays for the detection of pH, drugs (methamphetamine-like compounds), and intracellular bacterial enzymes (secondary amines) [106].

## 7. Biomarker Detection by Using Programmable p-DMF Devices

P-DMF devices, which have performances similar to those of other DMF devices, are novel microfluidic platforms because they can be used to manipulate small-volume droplets independently without the need for channels, pumps, and microvalves; thus, this type of microfluidic device, the p-DMF device, is most suitable for multi-step assay protocols, as well as for other single-step assay protocols [33,107]. In addition to their abilities to transport fluid and delay its flow, p-DMF devices can mix two or more samples effectively to allow homogeneous mixing between a sample and a reagent. Moreover, the p-DMF device is cost effective and disposable, which makes it more suitable for POCT than other DMF devices on glass or PCB. Although the use of p-DMF devices for chemical and biological assays is less popular than the use of other DMF devices fabricated on glass, plastic, and PCB, the use of p-DMF chips as platforms for detecting biomarkers has shown significant progress. 

**Figure 19:**
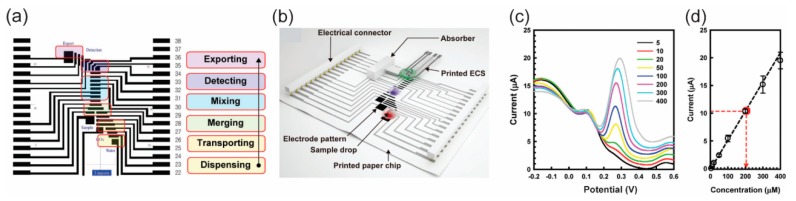
Electrochemical detection by using a p-DMF chip. (**a**) Programming sample delivery on the chip for assay protocols. (**b**) Schematic illustration of a p-DMF chip with an electrochemical sensor. (**c**) Electrochemical signal received from uric acid of various concentrations and (**d**) the calibration curve. Reprinted with permission from the authors of [28]. Copyright 2017 WILEY-VCH Verlag GmbH & Co. KGaA, Weinheim.

Simple assay protocols for colorimetric detection of glucose by using a p-DMF chip as a microfluidic platform was demonstrated [66]. In the detection, the p-DMF chip was mainly used for mixing the fluid samples and transporting the mixed sample to a p-CMF channel where the detection reagent was immobilized. Another group used a p-DMF chip as a microfluidic platform for sample preparation for protein digestion processes [108]. After protein digestion protocols (disulfide bond reduction, alkylation, buffering, and tryptic digestion) had been conducted using the paper chip, the samples were successfully analyzed by using a MALDI-TOF mass spectrometer to identify the proteins. A programmable, portable p-DMF chip was introduced for multiple electrochemical detections of biomarkers (Figure 19) [28]. Controlled by an Android smartphone via a wireless system, the p-DMF chip was programmed for multi-step assay protocols (Figure 19a). The fluid samples and reagents were transported, mixed, and washed in sequence on a paper chip for the detection of glucose, dopamine, and uric acid in human serum (Figure 19b–d). 

## 8. Challenges and Future Directions of Programmable Paper-Based Microfluidic Devices

Although paper-based microfluidic devices have made significant progress related to their fabrication method, operation technique, and application, they are still limited for analyzing challenging biomarkers such as cells and micro-nano bioparticles. Increasing the resolution of the p-CMF channels may allow such biomarkers to be studied by using p-CMF devices. Currently, because making very fine channels in p-CMF devices is more difficult than it is in polymer-based microfluidic devices, the latter devices are commonly used, instead of p-CMF devices, for the study of red blood cell deformation [109]. Moreover, being able to better control the characteristics of fluid transport such as the flow speed in p-CMF devices will pave the way for their application in a wide range of biomarker analyses. Unlike polymer-based microfluidic devices that are operated by external pumping systems, p-CMF devices are passively operated by capillary action. However, the flow of fluid in the p-CMF channel is weak and decreases with time. Because the surface of paper can be easily modified for chemical binding, p-CMF devices are commonly used for immobilizing enzymes, antibodies, and chemical reagents for chemical and biological assays [29].

Recently, the use of p-DMF devices for biomarker assays has increased significantly. With the recent development of fabrication methods, p-DMF devices can be fabricated easily and more affordably. Moreover, portable control systems and software for operation of the p-DMF devices are now well developed, so p-DMF devices are more flexible for fluid control. We encourage researchers to use p-DMF devices as microfluidic platforms for biomarker analyses. 

## 9. Conclusions and Outlook

Our review on the recent development of programmable paper-based microfluidic devices shows rapid progress in the fundamental understanding and engineering of devices for advanced fluid sample handling. In the near future, programmable paper-based microfluidic devices should become promising tools for screening patients for a wide range of diseases. Because the devices can be programmed for better control of fluidic sample transport, delay, valving, mixing, and merging, more and more diseases can be detected using a single- or a multi-step assay protocol with highly sensitive signal detection. Even though these programmable paper-based microfluidic devices have notable advantages in term of fluidic handling, they are currently more expensive than traditional paper-based microfluidic devices because they require additional modifications involving the use of functional materials and/or advanced engineering. If the price of assay detection is to become more affordable, their fabrication cost must be reduced because these devices are disposable. We encourage researchers to continue making contributions on the development of paper-based microfluidic devices, especially contributions regarding cheaper fabrication and controllability methods, so that they can be used for a wide range of applications. The use of existing programmable paper-based microfluidic devices to explore new methods for detecting new biomarkers is strongly recommended. 

## Figures and Tables

**Table 1:** Summary of published fabrication methods, materials, advantages, and disadvantages of for p-CMF devices.

Method [References]	Materials	Advantages	Disadvantages
Hot laminating [16]	Paraffin film	Simple and low-cost	Requires a cutter and hot-laminator
Wax printing [17,37,38,39]	Wax	Rapid and low-cost; no mask needed; programmable printing; mass production	Requires cure at high temperature
Screen printing [36,40]	Polydimethylsiloxane (PDMS); polymethylmethacrylate (PMMA)	Rapid and low-cost; can print with high viscosity ink	Requires cure at high temperature and screen mask
Photolithography [2,57]	Photoresist; octadecyltrichlorosilane (OTS)	High resolution; mass production	Requires clean room facilities
Direct handwriting [41]	Titanium oxide (TiO_2_)	Rapid and low-cost	Heavily depends on writers’ skill; difficult to mass produce
Inkjet printing [42,43,44]	Alkyl ketene dimer (AKD); commercial permanent marker ink	Customizable design; programmable printing	Requires low viscosity ink; nozzle clogging
Plotting [18,45,64]	PDMS; commercial permanent marker ink	Simple operation; low-cost	Requires a customized dispenser
Chemical vapor deposition (CVD) [46]	Trichlorosilane (TCS)	Rapid and simple process	Requires vacuum pump and mask
Cutting [20,21,22,47]	--	No hydrophobic agent is required; high resolution (laser cutter)	The resolution depends on the sharpness of a cutting blade; Expensive (laser cutter)
Layer-by-layer assembling [48,49,50,51,52,53]	R^F^SiCl_3_, wax, photoresist; tape	Can create 3D channel; multiple layer channels	Requires a cutter, tape, and holder
Molding [54]	Cellulose powder	No hydrophobic agents are required	Requires molding
Embossing [35,48,55,56]	R^F^SiCl_3_; wax; Paraffin film,	Can create 3D channel; simple and low-cost	Requires high pressure and molding
Laser treatment [57,58,59,60,61,62,63]	Octadecyltrichlorosilane (OTS); TiO_2_; photopolymer; hydrophobic so-gel	Can control surface energy of paper channel	Requires large area of hydrophobic coating on paper

**Table 2:** Summary of published fabrication methods, materials, advantages, and disadvantages of p-DMF devices.

P-DMF Components	Method [References]	Materials	Advantages	Disadvantages
**Conductive electrode arrays**	Inkjet printing [3,4,28]	Carbon nanotube (CNT); silver nanoparticle (AgNP)	Rapid; can produce thin electrodes; programmable printing	Nozzle clogging
	Screen printing [65,66]	Carbon; silver	Rapid and low cost; can print high viscosity ink	Requires a mask; produces thick electrodes
	Spraying [67]	Graphite	Simple and low cost	Requires mask; low resolution
	Micro-syringe dispensing [68]	AgNP	Can print high viscosity ink	Complicated setup
	Ballpoint pen printing [69]	AgNP	Simple and low cost; can produce thin electrode	Requires a customized ballpoint pen
**Dielectric-hydrophobic layer**	Chemical vapor deposition (CVD) [3,4,28]	Parylene-C/Teflon	High quality coating; controllable coating thickness	Requires clean room facilities; expensive
	Spin coating [68]	Polydimethylsiloxane (PDMS)/silicon oil	Simple process	Difficult to coat large area; excessive material waste
	Taping [66]	Adhesive tape/Nevosil	Simple and low cost	Depends on the quality of a commercial tape
	Wrapping [65,69]	Paraffin film; plastic wrap/silicon oil	Simple and low cost	Depends on the quality of a commercial wrap
